# Biomimetic virus-like mesoporous silica nanoparticles improved cellular internalization for co-delivery of antigen and agonist to enhance Tumor immunotherapy

**DOI:** 10.1080/10717544.2023.2183814

**Published:** 2023-02-27

**Authors:** Yuan Gao, Yingxi Zhang, Hong Xia, Yuqing Ren, Haibin Zhang, Siwen Huang, Meiju Li, Yongjun Wang, Heran Li, Hongzhuo Liu

**Affiliations:** a Wuya College of Innovation, Shenyang Pharmaceutical University, Shenyang, 110016, China; b School of Pharmacy, China Medical University, Shenyang, 110122, China

**Keywords:** Virus-like mesoporous silica nanoparticles, conventional mesoporous silica nanoparticles, nanocarrier drug delivery, co-delivery, immunotherapy

## Abstract

Nanocarrier antigen-drug delivery system interacts specifically with immune cells and provides intelligent delivery modes to improve antigen delivery efficiency and facilitate immune progression. However, these nanoparticles often have weak adhesion to cells, followed by insufficient cell absorption, leading to a failed immune response. Inspired by the structure and function of viruses, virus-like mesoporous silica nanoparticles (VMSNs) were prepared by simulating the surface structure, centripetal-radialized spike structure and rough surface topology of the virus and co-acted with the toll-like receptor 7/8 agonist imiquimod (IMQ) and antigens oocyte albumin (OVA). Compared to the conventional spherical mesoporous silica nanoparticles (MSNs), VMSNs which was proven to be biocompatible in both cellular and *in vivo* level, had higher cell invasion ability and unique endocytosis pathway that was released from lysosomes and promoted antigen cross-expression. Furthermore, VMSNs effectively inhibited B16-OVA tumor growth by activating DCs maturation and increasing the proportion of CD8^+^ T cells. This work demonstrated that virus-like mesoporous silica nanoparticles co-supply OVA and IMQ, could induce potent tumor immune responses and inhibit tumor growth as a consequence of the surface spike structure induces a robust cellular immune response, and undoubtedly provided a good basis for further optimizing the nanovaccine delivery system.

## 1. Introduction

Cancer immunotherapy, as a novel anticancer therapy, has attracted growing attention for its unique advantages, such as avoiding toxic effects, providing preventive action, inhibiting tumor metastasis, and preventing tumor recurrence (Finn, 2003; Cha et al., 2018; Zhou et al., 2020; Ren et al., 2021; Zhang et al., 2022; Xie et al., 2023). This body defense model involves a series of key steps, including the uptake and recognition of tumor-associated antigens (TAAs) by antigen-presenting cells (APCs), the presentation of the TAAs to T and B lymphocytes, and the initiation and activation of cellular and humoral immune responses. Among them, cytotoxic CD8^+^ T lymphocytes (CTLs) are crucial to anti-tumor efficacy through cytokine secretion and release of cytotoxic granules (Halle et al., 2017; Farhood et al., 2019; Shang et al., 2021). To our knowledge, the administration of TAAs, with or without adjuvants, has played an important role in tumor immunotherapy (Rosenberg et al., 2004). However, TAAs always display poor immunogenicity and inefficiency uptake effect of APCs, resulting in inability to produce an effective immune response (Kammer et al., 2007; Mody et al., 2013). To overcome these deficiencies, researchers work unremitting efforts to developing tumor vaccines that can induce sustained cellular and humoral immunity. To this end, nanocarrier antigen-drug delivery systems (Nano-DDS) have been proposed with a specific interaction to immune cells and intelligent delivery manners, which can effectively improve the antigen presentation efficiency and are helpful to achieve a successful immune procedure (Krishnamachari et al., 2011; Leleux & Roy, 2013; Wang et al., 2022). In general, the delivery process of Nano-DDS consists of a five-step CAPIR cascade, including circulation, accumulation, penetration, internalization, and intracellular release (Sun et al., 2017). Among them, cell internalization is a critical step affecting nanocarrier efficiency (Niu et al., 2013; Xia et al., 2009; Wang et al. 2017).

Encouraging, the diversification of nanocarrier in composition, morphology, size, shape, pore properties, surface/interface parameters (such as surface charge, surface chemistry, surface modification, and surface topography), etc., not only endows superiorities to multiple biological behaviors, but also is always consistent with functions. Meanwhile, these properties have proven to exert substantial effects on the behaviors of cells (including cellular uptake, endocytosis mechanism, and lysosomal escape), tissues, as well as organisms (distribution, metabolism, and excretion and biosafety). For example, different sized nanocarriers had different effects on lymph node transportation and specific cell interactions, in which 50–100 nm nanoparticles showed 175 times greater antigen delivery efficiency in follicular dendritic cells than 5–15 nm nanoparticles (Zhang et al., 2019). Meanwhile, cationic micelle vaccines were reported to induce potent humoral immune responses by promoting antigen uptake and the formation of germinal center formation (Luo et al., 2015). Until now, predecessors have exploited a larger number of Nano-DDS for the purpose of immunization, including liposomes, nanoemulsions and polymer micelle nanoparticles, etc., which have gradually become important members of immunologic adjuvants, substantially improved the immunotherapy outcomes, and in turn promoted the development of cancer vaccines (Jiang et al., 2018; Cheng et al., 2020; Wang et al., 2020). Unfortunately, there still remain the challenges of delivering their cargo to the desired sites due to the weak adhesion between nanocarriers and the cells, and the subsequent inadequate uptake by cells, which then easily captured and eliminated by the organism and causes the failure in immune response.

Once we talk about how to construct a Nano-DDS for immune purpose, the first teachers are the pathogens which can natively induce potent immune response. As one of the most ferocious pathogens, viruses are nanoscale entities exhibited centripetal-radialized spike structure and rough surface topology that can infect the organisms. They always have good cell invasion ability on account of the rough surface characteristics of their spike proteins, and are more easily penetrating through multiple physiological barriers. The current epidemic COVID-19 pandemic reminds us the power of viruses again, and the studies on the related viruses (SARS-CoV-2) also discover that the topological shape of viral spikes greatly influences their ability on attachment and invasion (Peng & Niemi, 2021; Li et al., 2022). Nanocarriers exhibiting viral topologies (Niu et al., 2013), called virus-like particle with the presence of spikes on their surface can be closely combined with the cell membrane, increase the structural defects of the cell membrane, and reduce the density of the lipid bilayer near the transmembrane transport process, thereby improving the absorption efficiency of the cell (Li et al., 2020). Inspired by the unique morphology of viruses, several works have been carried out recently to prepare virus-like particles, including viral nanoparticles, virus-like Fe_3_O_4_/Au@C-DOX-PEG (Zhao et al., 2021), and TiO_2_ microparticles decorated with nano-spikes (Wang et al., 2018). Especially, mesoporous silica nanoparticles which are easy to manufacture into a variety of shapes including a virus-like structure have attracted extensive attentions in biomedical fields as a consequence of their well-developed mesoporous structure, larger surface area, easy surface modification, stable physico-chemical properties, uniform size and good biosafety (Tang et al., 2012; Escriche-Navarro et al., 2022; Wang et al., 2022), and have been demonstrated as fascinating platforms for the delivery of therapeutic molecules.

**Scheme 1.**
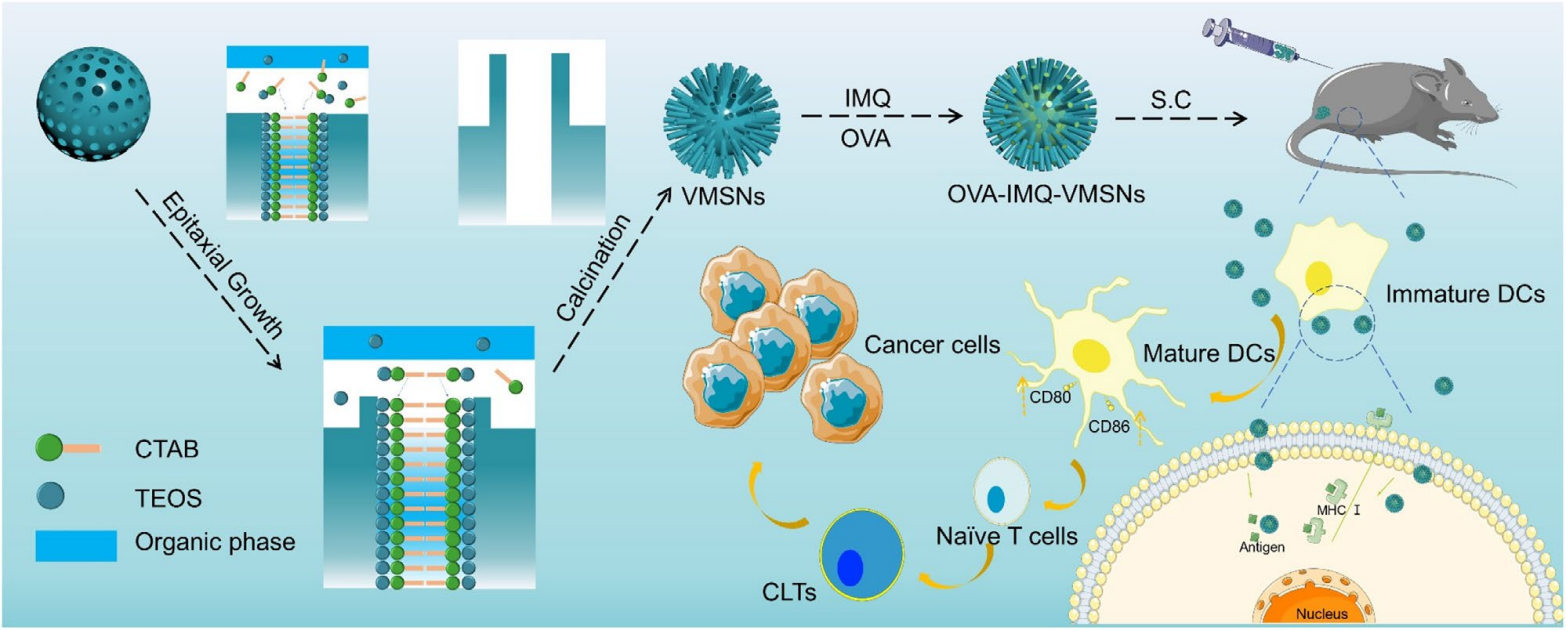
A simplified schematic diagram of OVQ-IMQ-VMSNs nanoparticles and their anti-tumor immune activation process.

In this study, a Nano-DDS based on uniform virus-like mesoporous silica nanoparticles (VMSNs) with inner mesoporous nanospheres surrounded by epitaxial vertical mesoporous nanotubes was designed and compared with the conventional mesoporous silica nanoparticles (MSNs) for immune activation and anti-cancer treatment (Scheme 1). Particularly, TLR 7/8 receptor agonist imiquimod (IMQ) was incorporated to VMSNs as an immunoregulatory model, and ovalbumin (OVA) was served as a model antigen. After vaccination, OVA-IMQ-VMSNs could be successfully internalized, and had a special uptake mechanism which facilitated endosomal escape of exogenous antigens for cross-presentation by APCs, leading to the present of antigen peptide effectively presented. As expect, OVA-IMQ-VMSNs inhibited the growth of B16-OVA tumor, promoted DCs maturation, and increased the proportion of CD8^+^ T cells. In general, with the assistance of virus-like morphology exhibiting good biosafety, the VMSNs-based Nano-DDS can significantly enhanced the ability of cells to uptake vaccines, improved the efficiency of vaccine delivery, and obtained satisfactory tumor therapeutic effect.

## 2. Materials and methods

### 2.1. Materials

Fluorescein 5 (6) – isothiocyanate (FITC, 95%), (3-Aminopropyl) triethoxysilane (APTES, 99.9%), Amiloride hydrochloride hydrate (Amil, 98%), Genistein (Geni, 98%), Chlorpromazine hydrochloride (CPZ, 98%), hexadecyltrimethylammonium bromide (CTAB), triethanolamine (TEA), and tetraethyl orthosilicate (TEOS) were purchased from Aladdin Reagent (Shanghai, China). IMQ was obtained from MCE (Shanghai, China). OVA were purchased from Sigma-Aldrich (Beijing, China). Mouse ELISA kits (TNF-α) for cytokine determination were provided by NeoBioscience Technology Co, Ltd, (Shenzhen, China). Fluorochrome-labeled anti-mouse monoclonal antibodies (FITC-CD3, PE-CD8, APC-CD4, FITC-CD80, PE-CD86, APC-CD11c) were bought from BioLegend Co, Ltd, (Beijing, China).

### 2.2. Animals

C57BL/6 mice were utilized according to the requirements and regulations of the Animal Ethics Committee of Shenyang Pharmaceutical University. All animal experiments were approved by the Animal Laboratory Ethics Committee of Shenyang Pharmaceutical University and performed under the guidelines for the care and use of laboratory animals. The number or ID of the ethics approval: SYPU-IACUC-C2021-5-12-110.

### 2.3. Synthesis of MSNs and VMSNs

In the synthesis of MSNs, a moderate amount of CTAB (0.8 g) was added to the reaction flask and stirred in the mixture of deionized water (100 mL), anhydrous ethanol (30 mL) and ammonia (0.8 ml) at room temperature. After 10 min, 2.5 mL TEOS was added, drop by drop, under a strong stirring, and stirred continuously for 4 h. After centrifugation for the removal of excess reactive fluid thereafter, the product was washed repeatedly with water and ethanol. The collected samples were then dried for 8 h in a vacuum at 45 °C, placed in a 100 mL 1% (v/v) methanol hydrochloric acid solution, refluxed for 12 h at 65 °C to remove the template, rinsed repeatedly with water and ethanol, and thoroughly dried.

For VMSNs, 1 g CTAB was added to 40 mL deionized water, followed by the addition of 0.18 g TEA. After gently stirring for 2 h, 20 mL n-hexane of TEOS solution of (20%, v/v) was added dropwise, and stirred for 48 h. After that, the flask was moved into oil bath of 90 °C and stirred for 24 h. The product was centrifugally collected, washed several times with water and ethanol, then dried for 8 h in a vacuum of 45 °C. The collected samples were placed in a solution of 100 mL 1% (v/v) methanol hydrochloric acid solution and refluxed for 12 h at 65 °C to remove the template CTAB. Samples were washed repeatedly with water and ethanol, and dried for 8 h in a 45 °C vacuum.

In the preparation of amine-modified VMSNs. 50 mg VMSNs was dispersed in 20 mL ethanol in which 500 μL APTES was added with stirring. After reflux reaction at 80 °C for 24 h, the product was collected by centrifugation, and alternating washed with ethanol and deionized water After thoroughly drying in a vacuum, the final product (VMSNs-NH_2_) was obtained. The preparation method of MSNs-NH_2_ is the same as VMSNs-NH_2_ described above.

### 2.4. Loading of OVA and IMQ in VMSNs and MSNs

In the drug loading process, IMQ was incorporated into VMSNs before loading OVA. First, different amounts of IMQ/VMSNs (0.025:1, 0.05:1, 0.1:1, 0.5:1, and 1:1 w/w) were stirred at room temperature for 24 h, centrifuged at 8000 rpm for 5 min, washed 3 times with deionized water, and collected the supernatants. IMQ encapsulation efficiency was used as an indicator to select the optimal drug carrier ratio, which was determined at 320 nm wavelength by UV − vis spectrometer. Besides, the encapsulation efficiency (EE, %) were calculated based on Eq. (1):

(1) $$\text{EE}\;\left(\%\right)\;=\;\frac{w_{1}-w_{2}}{w_{1}}\times 100$$

where W_2_ is free drug weigh, and W_1_ is added drug weight.

Afterwards, FITC was labeled with OVA via covalent binding. To be specific, 100 mg OVA and 5 mg FITC were lightly stirred in a carbonate buffer (pH 9.2) in the dark, and OVA-FITC was obtained after 24 h. Unreacted FITC was removed by dialysis. To load OVA or OVA-FITC, the selected IMQ-VMSNs were mixed with OVA or OVA-FITC (1 mL, 3 mg/mL on PBS) and vibrated in darkness at 4 °C for 1 h, allowing the mixture to adhere through electrostatic force. The final mixture was centrifuged for 5 min at 8000 rpm, washed 3 times with PBS. Meanwhile, OVA-FITC offloaded in the collected supernatants was measured using a microplate reader with 450 nm excitation wavelength and 500 nm emission wavelength. The OVA-FITC-IMQ-VMSNs were prepared and measured by the same procedure consistent with OVA. IMQ and OVA were also loaded into MSN in the same way.

### 2.5. Characterization of nanoparticles

The pore structure and surface morphologies of silica nanoparticles were characterized with TecnaiG2-F30 transmission electron microscope (TEM, FEI). The zeta potential and size of silica nanoparticles were measured by a dynamic light scattering (DLS) technique using Malvern Zetasizer NanoZS90. N_2_ adsorption desorption isotherms and pore size distributions were obtained at 77k using v-sorb 2800 P equipment. Structural information was confirmed by infrared spectroscopy (IR) with a wavelength range of 400 ∼ 4000 cm^−1^. For the determination of contact angle, 200 mg MSN and VMSN were compressed into tablets. A drop of water was carefully exposed to the tablet, and the wetting process was imaged by a high-resolution CCD digital camera. The photograph was then transmitted to a computer, and the contact angle of the sample was measured by a tangent method. Additionally, in order to study the stability and degradation trend of the carrier, the degradation rate of MSNs and VMSNs was studied at pH 7.4 PBS. The Weight loss (%) were calculated based on Eq. (2):

(2) $$\text{Weight loss }=\;\frac{w_{i}-w_{w}}{w_{i}}\times 100$$

where W_i_ is initial weight of carriers, and W_w_ is weight of residual samples.

### 2.6. In vitro release kinetics

In the OVA *in vitro* release study, OVA-IMQ-VMSNs/MSNs (1 mL, 4 mg/mL) were added to a dialysis bag (MWC 300 kDa) and vibrations were maintained in pH 7.4 PBS solution (0.01 M, 20 mL) and pH 5.5 MES solution (0.05 M, 20 mL) via swing bed (100 rpm). At a predetermined time, we took 1 mL of solution and added 1 mL fresh PBS or MES buffer. Cumulative OVA release were determined using emission wavelength microplate reader at 450 nm excitation wavelength and 450 nm emission wavelength. For the *in vitro* IMQ release study, OVA-IMQ-VMSNs/MSNs (1 mL, 4 mg/mL) were added in a dialysis bag (MWCO 10 kDa) and kept shaking by shakers (100 rpm) in pH 7.4 PBS (0.01 M, 20 mL) and pH 5.5 MES solution (0.05 M, 20 mL) at 37 °C. At a predetermined time, 1 mL of solution was collected and replaced by 1 mL fresh PBS or MES buffer. The cumulative IMQ release amount was measured at a wavelength of 320 nm by UV − vis spectrometer.

### 2.7. In vitro biosafety evaluation

*In vitro* cytotoxicity of RAW264.7 cells was determined by the MTT (methyl thiazolyl tetrazolium) assay. RAW264.7 cells (3.5 × 10^4^ cells/well) were initially seeded in 96-well plates in DMEM supplemented with 10% FBS, 1.5 g/L NaHCO_3_, 100 mg/L streptomycin and 30 mg/L penicillin and cultured in 5% cultured overnight in CO_2_ at 37° C. Thereafter, at 37 °C, VMSNs or MSNs solvents with concentrations ranging from 0 to 200 μg/mL were cultured for 24 h. Finally, 20 µL per well of MTT (5 mg/mL) in PBS solution was added to each well and continued to incubate at 37 °C for 4 h. After all solutions were removed, the purple formazan crystals were dissolved with 200 µL DMSO and the absorbance was recorded at 590 nm using a microplate reader. The Cell viability (%) were calculated based on Eq. (3):

(3) $$\text{Cell viability }(\%)=\;\frac{{OD}_{t}-{OD}_{b}}{{OD}_{c}-{OD}_{b}}\times 100$$

where OD_t_ is mean of absorbance value of medicine group, OD_b_ is mean of absorbance value of blank, and OD_c_ is mean absorbance value of control.

### 2.8. In vitro cellular uptake and the mechanism involved in the uptake process study

We loaded OVA-FITC into silica nanoparticles and measured the average intracellular fluorescence intensity by FACS analysis to quantitatively detect the uptake of different silica nanoparticles. RAW264.7 cells (3.0 × 10^5^ cells) were precultured in 12-well plates for 12 h and then incubated with free OVA-FITC plus IMQ, OVA-FITC-IMQ-VMSNs/MSNs (25 μg/mL OVA-FITC, 5 μg/mL IMQ) for 15 min, 30 min, 1 h, 2 h, 4 h and 8 h. After that, the cells were washed with PBS for more than 3 times to terminate ingestion and measured average intracellular fluorescence intensity and OVA-FITC positive RAW264.7 cells by FACS analysis. Absorption of VMSNs by cells 4 h after incubation were observed using CLSM under the same conditions.

With the aim of investigating the behavior of internalized silica nanoparticles, we used CLSM to observe the location of silica nanoparticles. RAW264.7 cells were incubated with free OVA-FITC plus IMQ, OVA-FITC-IMQ-VMSNs/MSNs (25 μg/mL OVA-FITC, 5 μg/mL IMQ) for 12 h after well plate growth. To determine the degree of co-localization of OVA and lysosomes, the images were treated with a CLSM after the cell nucleus was labeled by Hoechst and lysosomes stained by Lyso-Tracker Red.

To further investigate the cell uptake mechanism, RAW264.7 cells (3.0 × 10^5^ cells) were incubated in 24-well plates for 12 h. Before adding silica nanoparticles, the cells were incubated with the corresponding mechanism inhibitors for 1h, that is, chlorpromazine (CPZ, 20 μg/mL) was used to inhibit clathrin-mediated endocytosis, genistein (Geni, 2 μg/mL) was employed to suppress the caveolae-mediated endocytosis, and amiloride (Amil, 15 μg/mL) was served as the macropinocytosis inhibitor. The cells were then cultured with the OVA-IMQ-VMSNs/MSNs corresponding inhibitors for 6 h. Results were expressed as percentage uptake in the control group incubated with OVQ-IMQ-VMSNs/MSNs without inhibitors.

### 2.9. In vitro cytokine assays

Cytokine TNF-α was measured to determine whether the vaccine promoted cytokine secretion during APCs maturation. RAW264.7 cells (2.5 × 10^5^ cells/well) were seeded in 12-well plates overnight. After 8 h of culture, PBS was added as negative control, LPS (5 μg/mL) as positive control, and free OVA plus IMQ and OVA-IMQ-VMSNs/MSNs (20 μg/mL OVA, 5 μg/mL IMQ) were also introduced. Cell culture medium supernatant was collected and TNF-α concentrations in the supernatant determined by ELISA.

### 2.10. Evaluation of in vivo anti-tumor activity

The C57BL/6 mice (6 in each group) were subcutaneously injected with 1 × 10^6^ B16-OVA tumor cells. The tumor inoculation was treated with OVA-IMQ-VMSNs, OVA-IMQ-MSNs, free OVA plus IMQ (containing 20 μg of IMQ and 80 μg of OVA) and PBS 100 μL on day 5 and again on day 10 and 15, respectively. Survival, tumor size and mice weight were monitored. Tumor volume digital calipers measured. The tumor volume (mm^3^) were calculated based on Eq. (4):

(4) $$\text{Tumor volume}=\frac{a\times b^{2}}{2}$$

where a is length, and b is width.

### 2.11. Immune activation in tumor-bearing mice

The tumorigenic mice were euthanized three days after the last treatment. Isolated mouse lymph nodes were stained with fluorescent antibodies (FITC anti-mouse CD80, PE anti-mouse CD86, APC anti-mouse CD11c) into cell suspension. CD11c^+^CD80^+^ and CD11c^+^CD86^+^ expression were measured by flow cytometry.

The mouse spleen was grated, sifted and treated with erythrocyte lysate to obtain cell suspension. Fluorescent antibodies (FITC anti-mouse CD3, PE anti-mouse CD8, APC anti-mouse CD4) were added to the cell suspension. CD3^+^CD8^+^ and CD3^+^CD4^+^ expression were measured by flow cytometry.

### 2.12. TUNEL and immunofluorescence (IFC) assays

Tumor tissue sections of mice were stained to study the effects of the nanovaccine on proliferation of CD8^+^ T cells, CD4^+^ T cells, M1 macrophages and M2 macrophages in tumor tissues. Analysis of tumor cell apoptosis was performed by TUNEL method.

### 2.13. Bio-safety evaluation

To examine the *in vivo* safety, subcutaneous injection of MSNs and VMSNs were performed on 0, 5, and 10 days, respectively, and control group was injected with saline. After 14 days, blood routine and biochemical blood tests were performed. Biopsies of the skin, heart, liver, spleen, lungs and kidneys were taken to assess the vaccine’s biosafety.

### 2.14. Statistical analysis

All data were expressed in this article as mean result ± standard deviation (SD). Data were analyzed for statistical significance using one-way variance analysis. p < 0.05 was considered statistically significant. Statistical significance was defined as *p < 0.05, **p < 0.01, and ***p < 0.001, ****p < 0.0001. All statistical analysis were performed using GraphPad Prism 8 software.

## 3. Results and discussion

### 3.1. Preparation and characterization of OVA-IMQ-VMSNs

**Figure 1.**
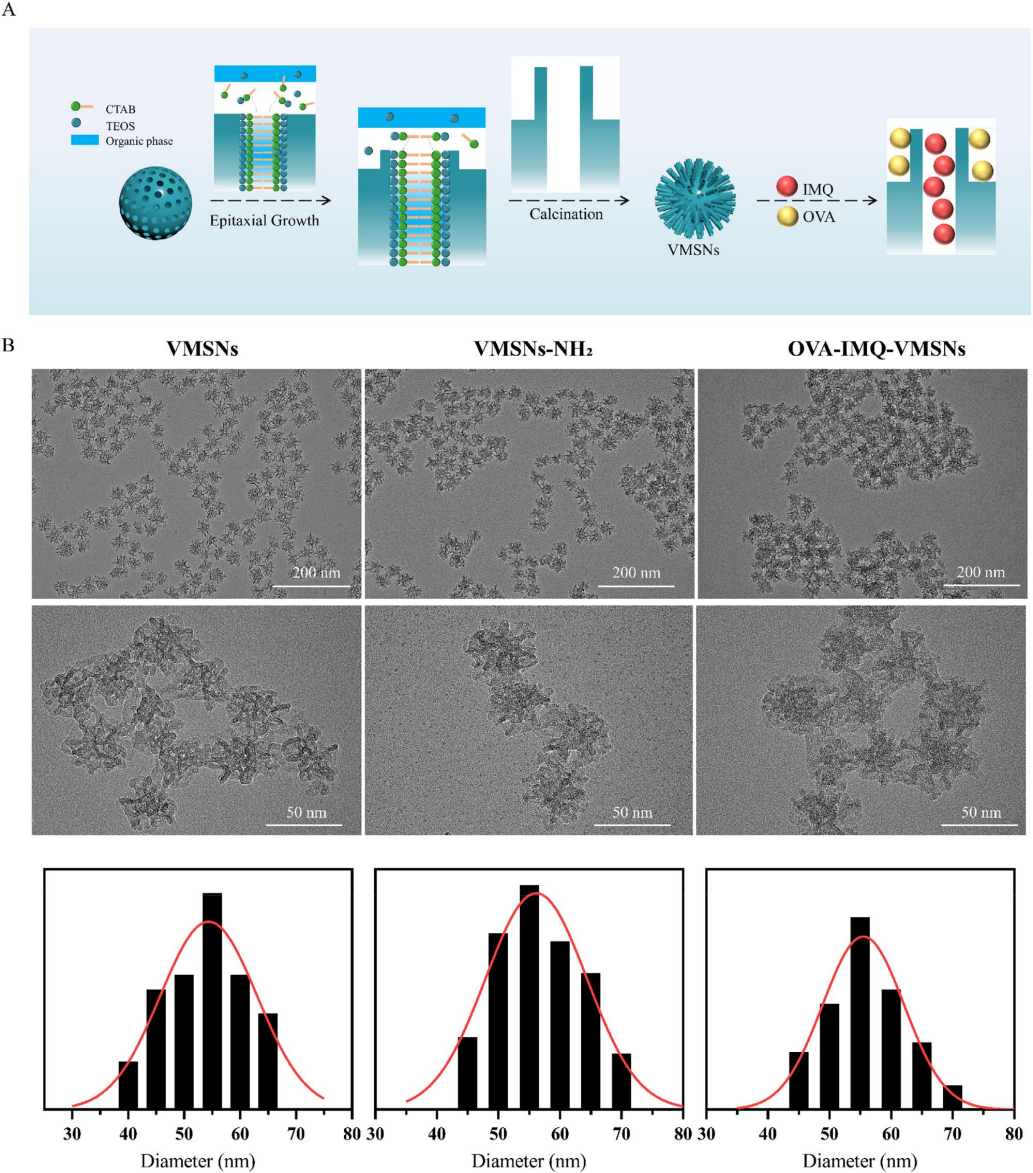
Structure of virus-like mesoporous silica nanoparticles. (a) Schematic illustration of the VMSNs formation and drug loaded process. (b) TEM image of VMSNs, VMSNs-NH_2_, OVA-IMQ-VMSNs.

**Figure 2.**
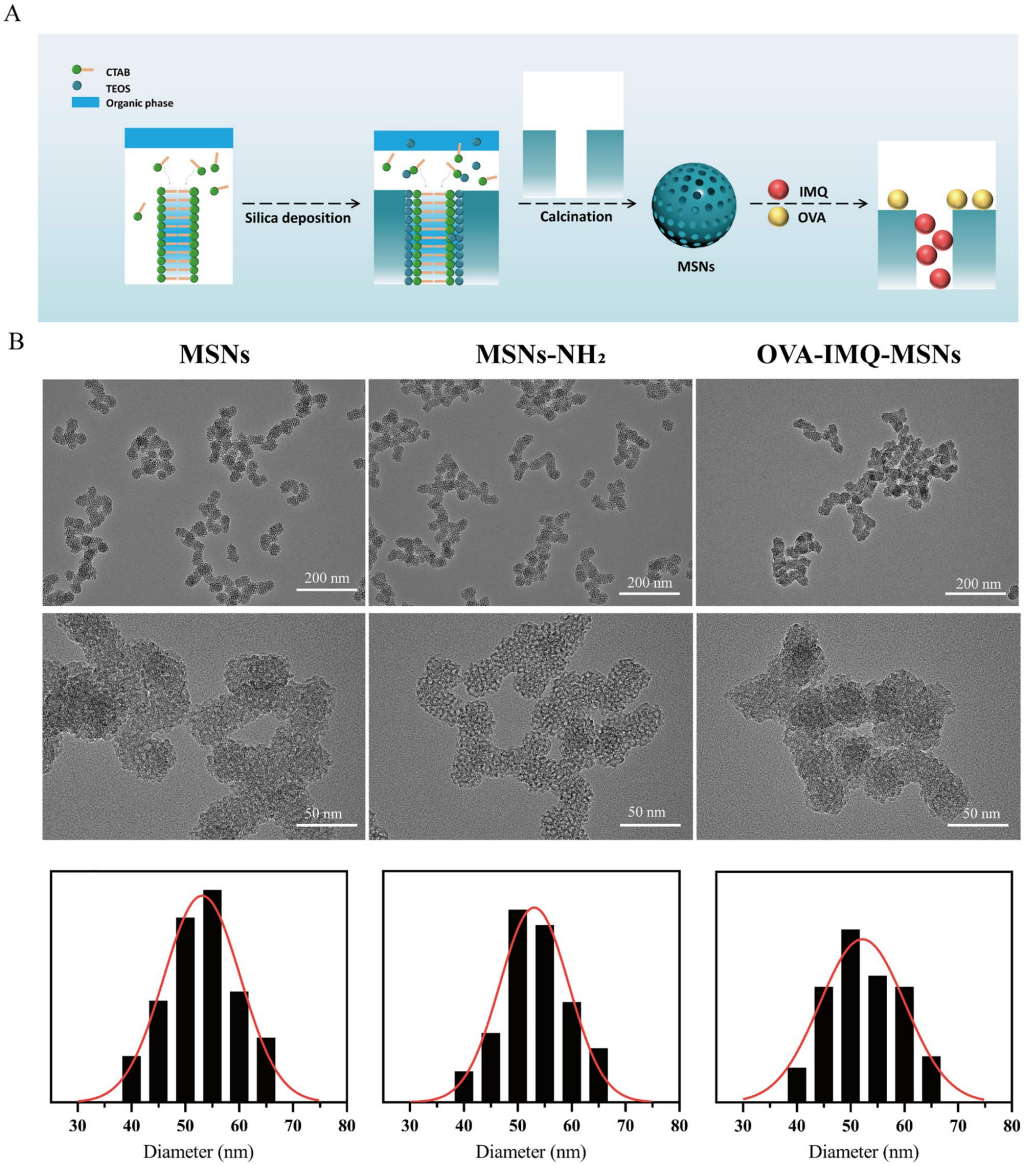
Structure of conventional mesoporous silica nanoparticles. (a) Schematic illustration of the MSNs formation and drug loaded process. (b) TEM image of MSNs, MSNs-NH_2_, OVA-IMQ-MSNs.

**Figure 3.**
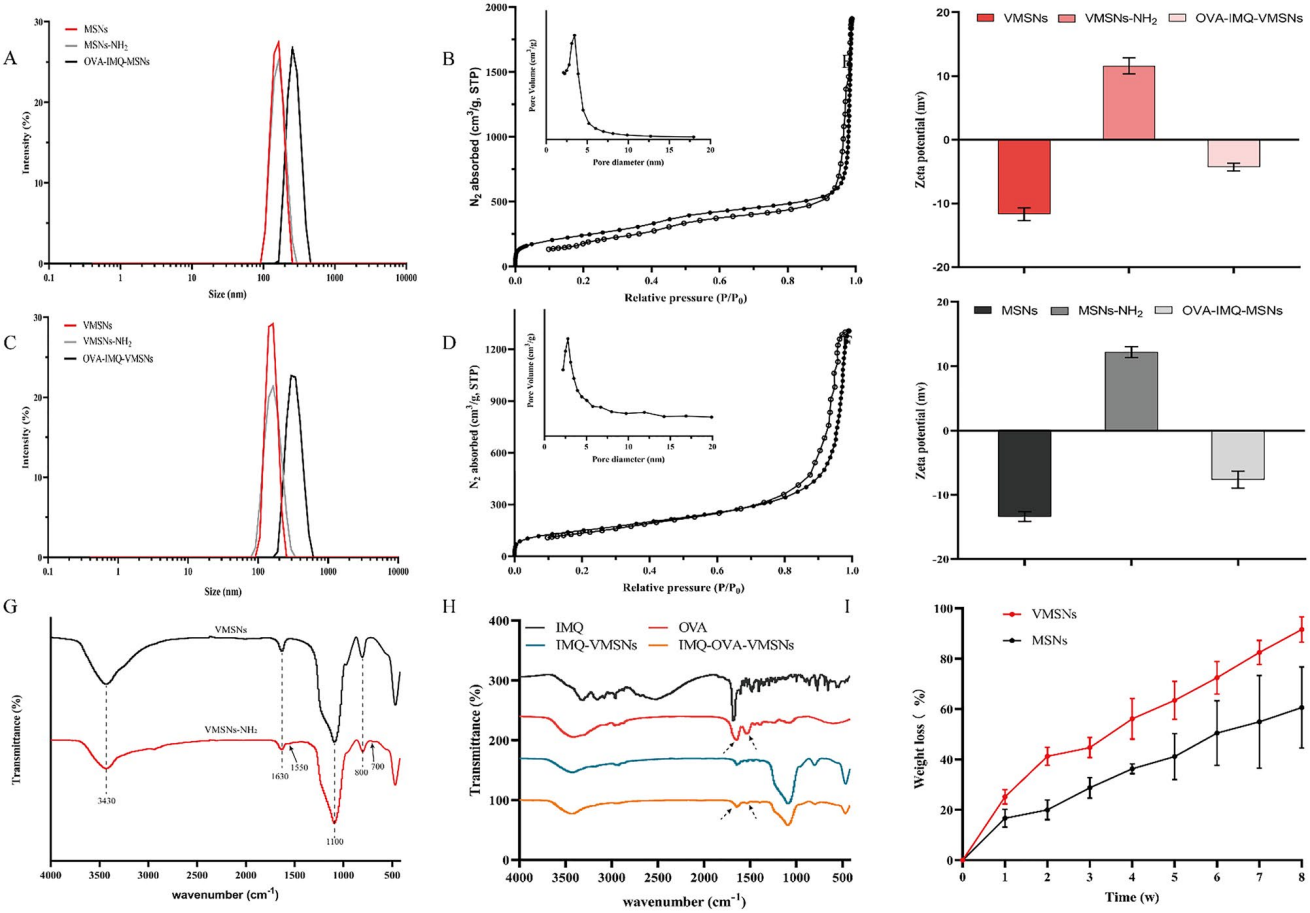
Characterization of silica nanoparticles. (a) Dynamic light scattering of VMSNs, VMSNs-NH_2_, OVA-IMQ-VMSNs. (b) Nitrogen adsorption-desorption isotherms and pore size distribution profile of VMSNs. (c) Dynamic light scattering of MSNs, MSNs-NH_2_, OVA-IMQ-MSNs. (d) Nitrogen sorption isotherms and the corresponding pore size distribution obtained from adsorption branch of MSNs. (e) Surface zeta potential of VMSNs, VMSNs-NH_2_, and OVA-IMQ-VMSNs. (f) Surface zeta potential of MSNs, MSNs-NH_2_, and OVA-IMQ-MSNs. (g) FTIR spectra of VMSNs and VMSNs-NH_2_. (h) FTIR spectra of IMQ, OVA, VMSNs loaded IMQ and VMSNs loaded IMQ plus OVA. (i) Degradation rate of VMSNs and MSNs (pH 7.4).

**Figure 4.**
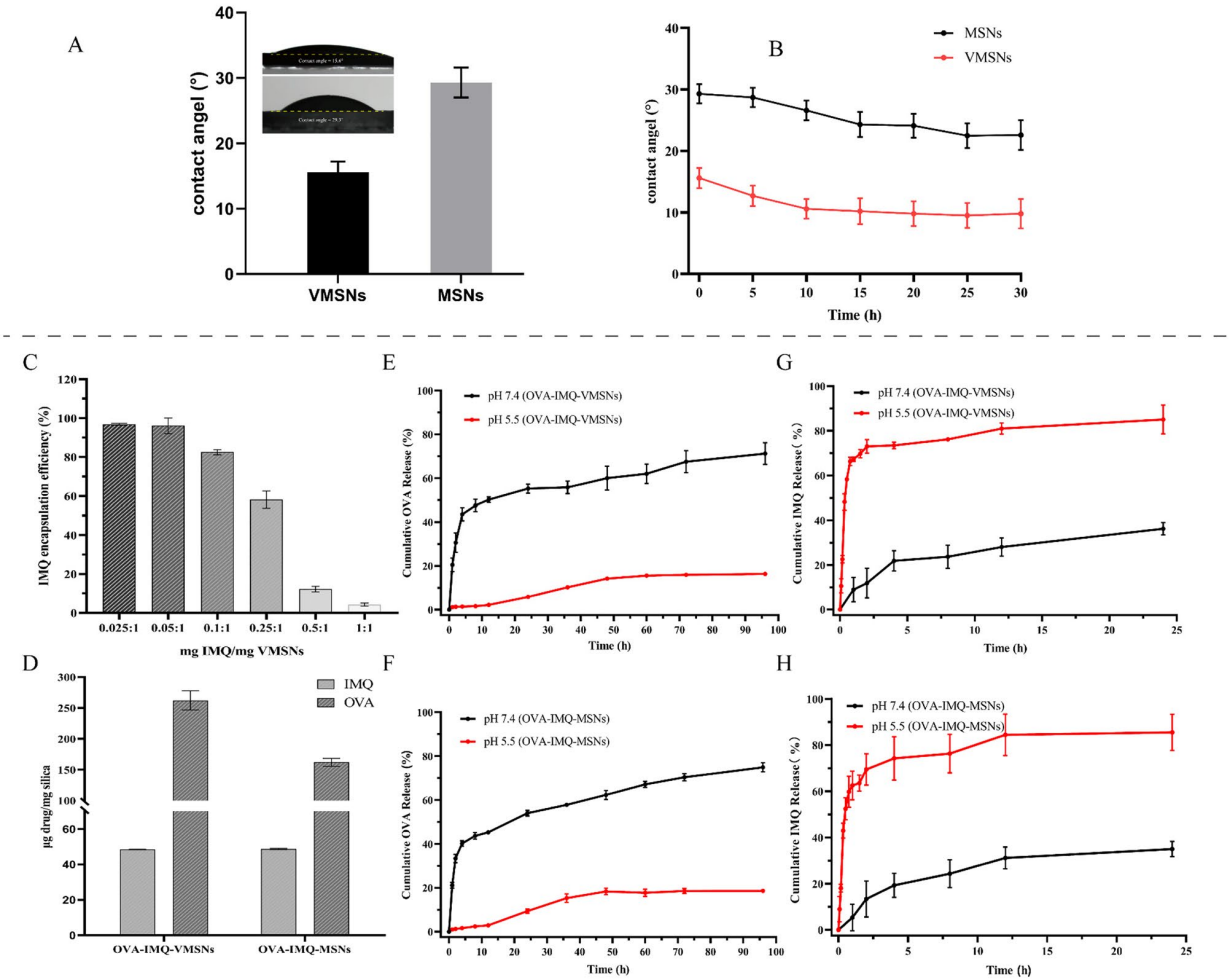
Prescription screening and *in vitro* release of silica nanoparticles. (a) Initial contact angles of VMSNs and MSNs. (b) Variation of contact angle with time. (c) IMQ encapsulation efficiency of different amounts of IMQ/VMSNs. (d) Loading amounts in VMSNs and MSNs. (e) *In vitro* OVA and IMQ release behavior in PBS (pH 7.4) and (g) MES buffer (pH 5.5) of OVA-IMQ-VMSNs. (f) *In vitro* OVA and IMQ release behavior in PBS (pH 7.4) and (h) MES buffer (pH 5.5) of OVA-IMQ-MSNs (*n* = 3).

First, to imitate the unique surface morphology of virus, uniform VMSNs with inner mesoporous nanospheres surrounded by epitaxial perpendicular mesopore nanotubes was successfully synthesized via a novel single micelle epitaxial growth approach in a low-surfactant-concentration oil/water biphase reaction system, using CTAB as a structural template and TEOS as a precursor (Figure 1a). As presented in the TEM image (Figure 1b), VMSNs had a unique virus-like morphology with uniform particle size of ∼ 60 nm and exhibited plenty of spikes on their surface. In contrast, conventional MSNs were uniform ∼ 60 nm nanospheres with smooth surface topology (Figure 2b). DLS measurements also revealed a stable particle system with a narrow particle size distribution (Figure 3a,c), which showed a mean value of 133.0 and 140.2 nm (hydrodynamic diameter) for VMSNs and MSNs, respectively. The particles measured in solution using DLS method had the larger hydrodynamic radius and some additional agglomeration effects, thereby resulting in the higher value than the actual size. This might be why TEM and DLS measured different sizes (Wang et al., 2021). Moreover, the pore size and pore volume of VMSNs were determined by N_2_ sorption (Figure 3b), which confirmed uniform mesoporous structure of VMSNs and MSNs evidenced by the typical type IV pattern isotherms with clear condensation steps. The pore size distribution curves showed a single 3.45 nm peak and a single 2.80 nm peak for VMSNs and MSNs, respectively (Figure 3b,d). Besides, The Brunauer − Emmett − Teller (BET) surface area and pore volume of VMSNs and MSNs were measured to be 524.78 and 882.48 m^2^ g^−1^, 2.02 and 2.95 cm^3^ g^−1^, respectively (Table 1), suggesting sufficient nanospace for drug loading. Particularly, wettability is a very important interface property which is closely related to *in vitro* and *in vivo* functions (Hu et al., 2020). The wettability of VMSNs as an effective drug carrier was investigated. According to Figure 4a, VMSNs had a contact angle of 15.6 ± 1.6° and was less than MSNs (29.3 ± 2.3°), indicating that VMSNs was rougher due to its unique structure, which would facilitate the carrier-cell contact and uptake. As time changes, the contact angle of VMSNs and MSNs became smaller. (Figure 4b) Additionally, to ensure their stability and structure foundation as drug carriers, the degradation study was performed. At pH 7.4, the degradation process of VMSNs and MSNs was similar, both of which experienced mild degradation processes. It showed that VMSNs have good stability in the short term, and can be metabolized without burden to the body in the long run (Figure 3i).

**Table 1.** Pore size, pore volume, and surface area of VMSNs and MSNs.

	BET surface area (m^2^/g)	Pore Volume (cm^3^/g)	Pore size (nm)
VMSNs	524.78	2.02	3.45
MSNs	882.48	2.95	2.80

To construct a cancer vaccine using VMSNs, two drugs, OVA and IMQ, both of which were important components in the formulation of cancer vaccine were introduced to the system. To be specific, the OVA protein was used as a model antigen, while IMQ was a toll-like receptor 7/8 (TLR 7/8) immune response agonist. Particularly, as a biological macromolecule drug, OVA was difficult to enter the pores of mesoporous materials, so adsorption method was employed to bound OVA on the surface of VMSNs. Under physiological conditions, OVA showed a slight negative charge. Therefore, in order to enhance the drug loading amount, we treated VMSNs and MSNs with ammonia. After the amine modification, FTIR spectra of the VMSNs-NH_2_ showed the existence of N − H characteristic peak at 1550.0 and 700.0 cm^−1^ (Figure 3g), confirming the successful grafting of amine groups. The surface zeta potential of VMSNs-NH_2_ was shifted from −11.7 mV to 11.6 mV and MSNs-NH_2_ was increased from −13.4 mV to 12.2 mV (Figure 3e). The surface of MSNs and VMSNs had a large amount of silicon hydroxyl groups, which were deprotonated in an aqueous solution. The particle size increased slightly, with 150.8 nm for VMSN-NH_2_ and 163.7 nm for MSNs-NH_2_ (Figure 3a,c). Besides, VMSNs-NH_2_ and MMSNs-NH_2_ displayed no significant variation on TEM images compared to VMSNs and MMSNs, and the typical surface topology also remained unchanged (Figures 1b and 2b).

Furthermore, different amounts of IMQ/VMSNs (0.025:1, 0.05:1, 0.1:1, 0.5:1 and 1:1 w/w) were stirred at room temperature for 24 h, and the encapsulation efficiency was used as the index to select the optimal prescription. As depicted in Figure 4c, the encapsulation efficiency of IMQ reduced gradually with the increasing proportion of IMQ. When IMQ/VMSNs were 0.025:1 and 0.05:1, the encapsulation efficiency was similar, and 0.05:1 was selected as the optimal proportion to achieve a higher drug loading amount. After IMQ load, OVA was bounded through electrostatic adhesion by vibrating at 4 °C for 1 h with aminated VMSNs. The final drug load of VMSNs was 48.45 μg IMQ/mg silica and 262.15 μg OVA/mg silica. The drug load of MSNs was 48.69 μg IMQ/mg silica and 161.99 μg OVA/mg silica (Figure 4d). The results indicated that MSNs had a slightly lower ability to load OVA than VMSNs. It was observed under TEM that the OVA-IMQ-VMSNs after drug loading did not destroy the original viral morphology, and OVA-IMQ-VMSNs were more likely to agglomerate compared to that before drug loading. The situation of OVA-IMQ-MSNs is similar with OVA-IMQ-VMSNs (Figures 1b and 2b). Meanwhile, DLS measurements of OVA loaded VMSNs and MSNs in aqueous solutions demonstrated a shift to higher hydrodynamic sizes of ∼ 291.9 and ∼ 231.4 nm (Figure 3a,c), which could be explained by the attachment of the protein biological macromolecule drug as well as a small agglomeration effect. This phenomenon can also be observed under TEM. FTIR analysis further confirmed that IMQ and OVA were effectively loaded into VMSNs. When IMQ was loaded into VMSNs, the stretching vibration peaks of belonging to the benzene ring in the infrared spectrum almost disappeared, which further proved that IMQ were successfully loaded into the pores of VMSNs. When OVA was loaded into VMSNs, the infrared characteristic peak of OVA can still be seen. It indicates that OVA is loaded on the surface of VMSNs by adsorption (Figure 3h). Additionally, OVA-IMQ-VMSNs and OVA-IMQ-MSNs showed negative zeta-potential charges of −4.3 and −7.7 mV, respectively (Figure 3e). The reversal of zeta-potential again also proved the success of drug loading.

### 3.2. OVA and IMQ in vitro release behavior

We then studied the antigen and adjuvant release behaviors from vaccines with different pH values (7.4 and 5.5) to simulate the cytoplasm mimetic environment and endosome/lysosome of APCs. First, antigen release behavior in OVA-IMQ-VMSNs and OVA-IMQ-MSNs both showed a slow upward trend. To be specific, OVA-IMQ-VMSNs exhibited total release up to ∼ 55.24% within 24 h and ultimately ∼ 71.20% within 96 h under pH 7.4 PBS. However, antigen release behavior of OVA-IMQ-VMSNs in pH 5.5 MES buffer only had a total release of ∼ 5.77% and ∼ 16.29% within 24 and 96 h, respectively (Figure 4e). The result indicated that antigens showed both higher release rate and release amount in a neutral environment (the cytoplasm mimetic), which would facilitate vaccine release into the cytoplasm when released from the lysosome (Zhang et al., 2019). Next, IMQ exhibited an acidic pH-responsive drug release pattern from OVA-IMQ-VMSNs, in which ∼ 67.26% of IMQ was released in the first 1 h and ∼ 85.07% over 24 h under pH 5.5. However, when the pH was raised up to 7.4, only ∼ 8.98% was released in the first 1 h, followed by a slower release (∼ 36.25%) over 24 h (Figure 4g). At an acidic environment (pH 5.5), IMQ could release rapidly, which would promote more IMQ binding to TLR 7/8 in the microenvironment of endoplasmic/lysosomal cells (Aichhorn et al., 2017). It should be noticed that, the release behaviors of antigen and adjuvant from OVA-IMQ-MSNs were essentially the same as those of OVA-IMQ-VMSNs, with slight differences in release amounts (Figure 4f,h).

### 3.3. Cellular uptake efficiency of vaccines and lysosomal escape of antigens

**Figure 5.**
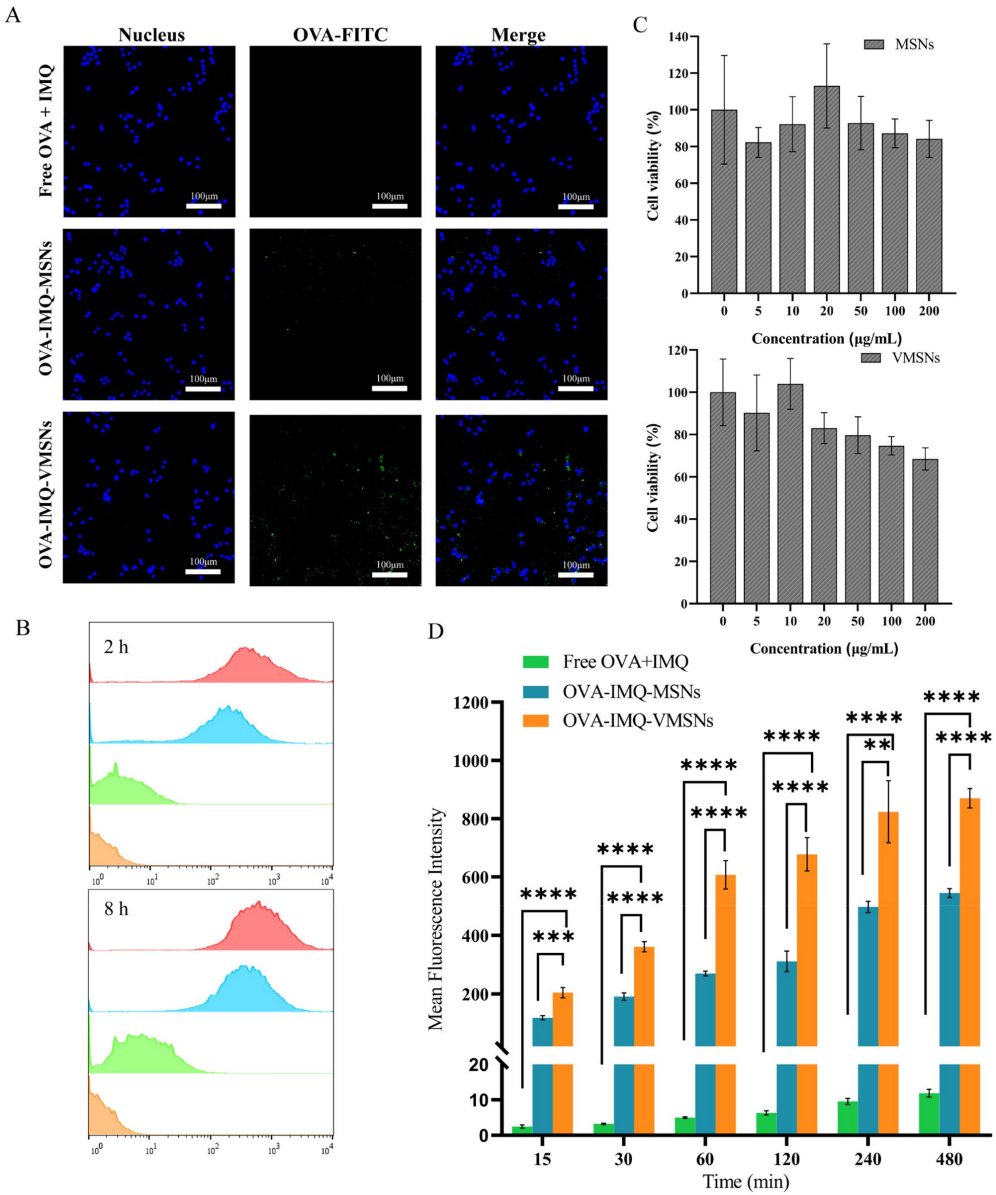
The effect of silica nanoparticles on cellular toxicity, uptake and cytokine secretion of RAW264.7 cells. (a) CSLM florescence image of RWA264.7 cells cultured with free OVA plus IMQ and OVA-IMQ-VMSNs/MSNs during 4h. (b) Flow cytometry analysis of OVA-FITC positive cells which cultured with OVA-IMQ-VMSNs/MSNs. (c) Cytotoxicity of VMSNs and MSNs at various concentrations (*n* = 6). (d) Flow cytometry analysis means fluorescence of cells cultured with OVA-IMQ-VMSNs/MSNs with different times (*n* = 3).

The vaccine was first tested for toxicity before undergoing a series of *in vitro* and *in vivo* experiments. MTT assay revealed that nanoparticles showed good biocompatibility to cells, which had low cytotoxicity only at high concentrations (Figure 5c). Meanwhile, since cellular uptake performance of APCs largely determined the efficiency of vaccines, the cellular uptake efficiency of vaccine was then evaluated by CLSM and flow cytometry (Lin et al., 2010). Herein, MSNs and VMSNs loaded with FITC-labeled OVA were incubated with RAW264.7 cells for detection with free OVA plus IMQ as a reference. Flow cytometry analysis discovered that cells variational internalized nanoparticles with different morphologies, in which the OVA-IMQ-VMSNs displayed dramatically higher uptake level than OVA-IMQ-MSNs and free OVA plus IMQ (Figure 5b). Meanwhile, as can be seen in Figure 5d, the degree of RAW264.7 cells uptake increased with time. Compared with OVA-IMQ-MSNs, OVA-IMQ-VMSNs also increased the MFI level of OVA-FITC in RAW264.7 cells by about 2-fold, and which was much greater than that of free drugs (Figure 5d). Corresponding CLSM image results were consistent with the flow cytometry findings (Figure 5a), and clearly demonstrated that the VMSNs could enter macrophages in large quantities. In should be noticed that, VMSNs and MSNs exhibited similar size distribution, good monodispersity, and excellent stability on the basis of the previous findings (DLS, zeta-potential and TEM measurements, Figures 1–3). Therefore, it could be reasonable assumed that the differences in cellular uptake property were mainly attributed to the surface morphology.

**Figure 6.**
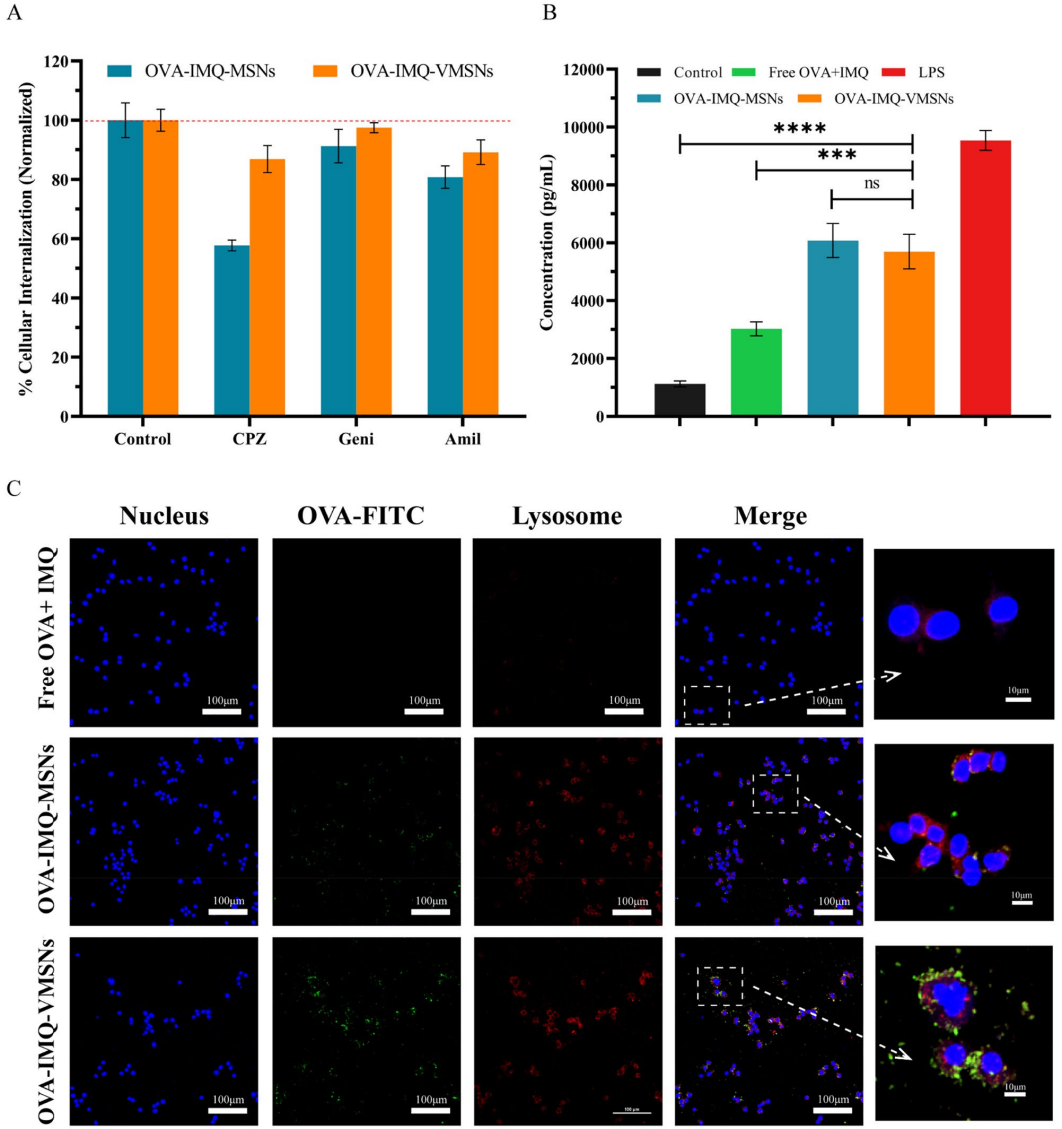
Uptake of different formulations by RAW264.7 cells. (a) Cells were incubated with different inhibitors. Percent internalization was normalized to particle internalization in the absence of inhibitors. (b) Secreted TNF-α from RAW264.7 cells measured by ELISA. (c) Localization of different formulations at endosomes/lysosomes in RAW264.7 cells observed by CSLM. OVA-FITC (green color), the nucleus labeled with Hoechst (blue color) and lysosomes labeled with Lyso-Tracker (red color).

With the aim of further exploring the internalization pathways, the vaccines were treated with cells in the presence of endocytic inhibitors including CPZ, Geni, and Ami, respectively. Interestingly, these inhibitors significantly all decreased the uptake of the nanoparticles, suggesting the involvement of multiple endocytosis mechanisms, including clathrin-mediated endocytosis, caveolae-mediated endocytosis, and micropinocytosis. As shown in Figure 6a, the cellular uptake of the MSNs and VMSNs was prohibited to a higher degree after incubated with CPZ, and suppressed to a slighter degree in the presence of Geni and Ami, indicating a dominant clathrin-mediated endocytosis. Specially, a larger inhibition proportion of MSNs was observed utilized CPZ (relevant to clathrin-mediated endocytosis) than VMSNs. Actually, the cellular uptake of the MSNs was dropped to a lower level in the presence of inhibitors, while none of the above specific chemical inhibitors led to markedly inhibition of cellular uptake to VMSNs. Therefore, we speculated that clathrin/caveolae-independent endocytosis, which was known to render the formation of primary endosomes and consequently form late endosomes and lysosomes, most probably played a crucial role in the uptake of the VMSNs (Wang et al., 2011). This clathrin/caveolae-independent endocytosis unique endocytic pathway of the VMSNs may be responsible for its high endocytosis efficiency, and may help the VMSNs escape from lysosomes.

After uptake by APC, the location of the antigen in cells also affects the route of the immune response. To our knowledge, endosomal escape promotes efficient antigen delivery to cytoplasm, enhances cross-expression of exogenous antigen to MHC I molecules, and activates CD8^+^ T cells to elicit robust CTLs (Keller et al., 2014; Liu et al., 2015; Duan et al., 2017; Day et al., 2021). The antigen uptake and intracellular distribution of nanoparticles were then observed by CLSM. According to Figure 6c, the cells treated with free FITC-OVA (green) or OVA-IMQ-MSNs coincided with labeled lysosomal membranes (red), indicating that OVA was mainly within the lysosomes. In contrast, after OVA-IMQ-VMSNs treatment of cells, the green fluorescence was broadly distributed in the cytoplasm other than coincided with the red fluorescence of labeled lysosomal membrane, suggesting that the vaccine could escape from the lysosomes and achieve high efficiency of antigen cytoplasmic delivery. The efficient lysosomal escape ability of OVA-IMQ-VMSNs may be related to its unique clathrin/caveolae-independent endocytosis pathway, which also confirmed our previous conjecture.

### 3.4. Evaluation of immune response in vitro

IMQ is a toll-like receptor 7/8 agonist that stimulates APCs to secrete cytokines (Lin et al., 2017). Next, we tested cytokine TNF-α to verify whether VMSNs delivery IMQ can stimulate cytokine secretion during APCs maturation. As shown in Figure 6b, OVA-IMQ-VMSNs/MSNs could induce cells to produce more TNF-α. This indicated that adjuvant-loaded MSN and VMSN both stimulate cells to produce a stronger immune response *in vitro* because of increased cell uptake.

### 3.5. Evaluation of in vivo antitumor effects

**Figure 7.**
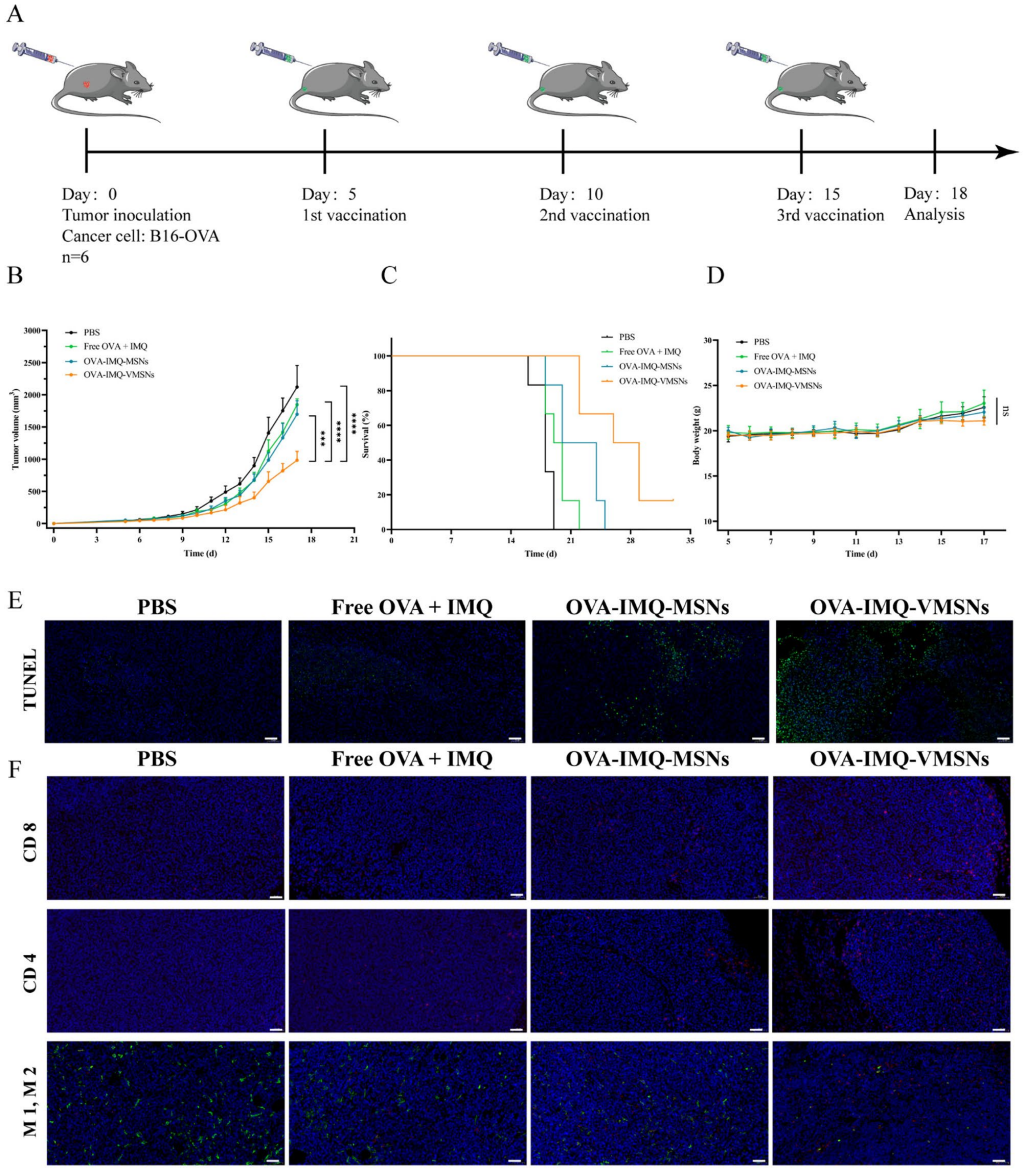
Therapeutic effects of nanovaccine against established B16-OVA tumor model. (a) Schedule of against established B16-OVA tumor model vaccination. Mice were vaccinated with PBS, a mixture of OVA and IMQ, OVA-IMQ-MSNs and OVA-IMQ-VMSNs (OVA 80 μg and IMQ 20 μg). (b) Tumor growth curve, (c) mouse survival after various treatments and (d) Bodyweight changes of mice after different treatments (*n* = 6). (e) TUNEL assay of the tumor. (f) Representative immunofluorescence staining for CD8 (red), CD4 (red), M1 macrophages (red) and M2 macrophages (green) of tumor sections obtained after aforementioned treatments, while nucleus was stained with DAPI (blue). Scale bar: 50 μm.

To investigate whether OVA-IMQ-VMSNs immunotherapy can exert systemic antitumor effects, C57BL/6 mice were subcutaneously implanted with 1 × 10^6^ B16-OVA cells in the back. According to the scheme of inhibiting subcutaneous B16-OVA tumor (Figure 7a), the mice were injected subcutaneously of PBS, a mixture of OVA plus IMQ, OVA-IMQ-MSNs and OVA-IMQ-VMSNs at the base of the tail at the 5th day of tumor inoculation at the injection dose of 80 μg OVA and 20 μg IMQ, and the above drugs were re-injected at the 10th and 15th days, respectively. In Figure 7b, it was found that the tumor volumes in free OVA plus IMQ, OVA-IMQ-MSNs and OVA-IMQ-VMSNs groups were suppressed to different degrees. Particularly, tumor growth inhibition of the OVA-IMQ-VMSNs group was more potent than that of the OVA-IMQ-MSNs group and free OVA and IMQ. The survival time of mice in OVA-IMQ-VMSNs group was also notably prolonged (Figure 7c). The results showed that VMSNs had high cellular uptake efficiency and unique uptake mechanism to promote antigen uptake and cross-expression, thus promoting immune response to OVA-expressed tumor cells. In addition, the mice’s weight was monitored during the experiment and no significant weight loss was observed, confirming that the vaccine was not systematically toxic to mice (Figure 7d).

### 3.6. Assessment of immune activation in vivo

**Figure 8.**
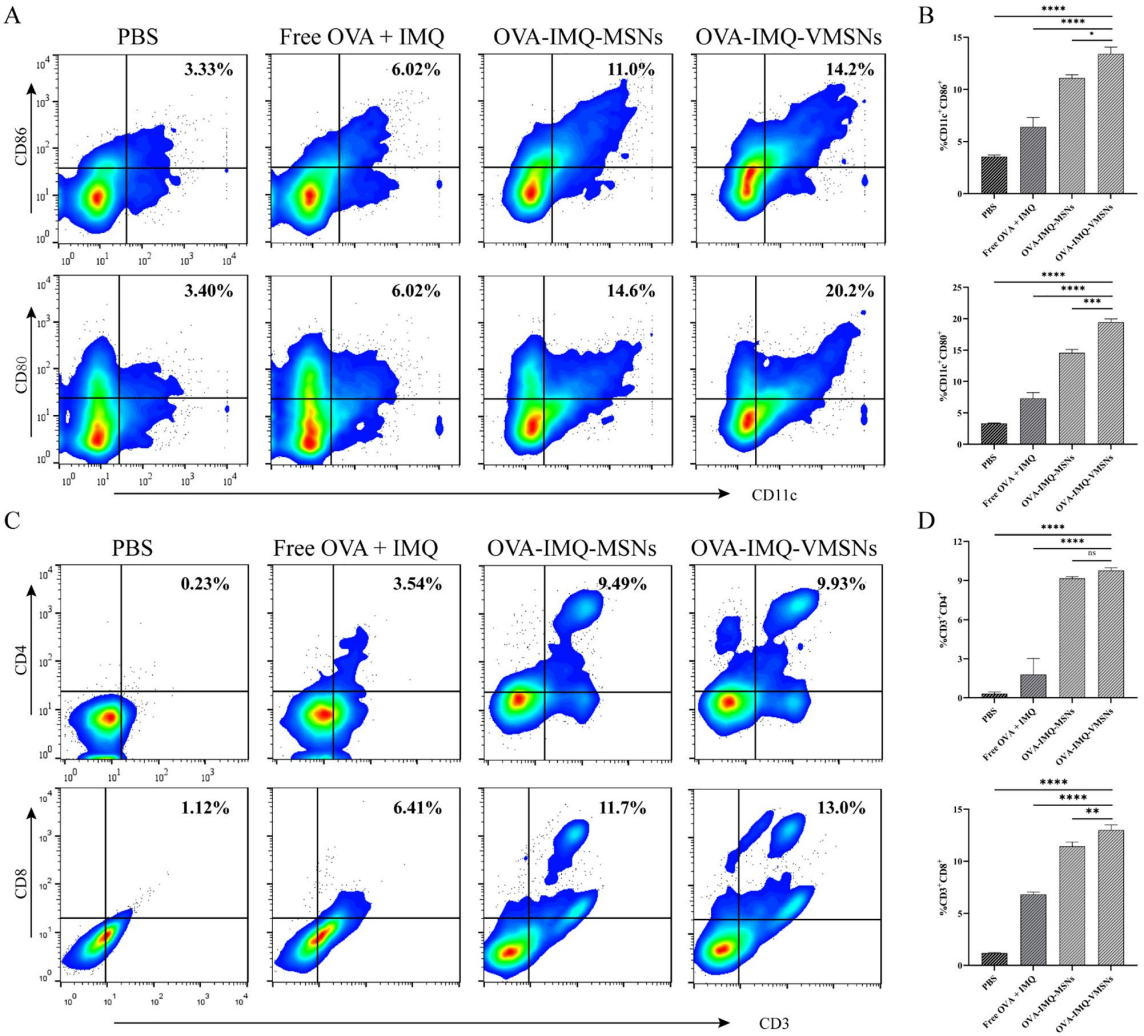
Evaluation of immune response *in vivo*. The tumor-bearing mice were euthanized three days after the last immunization, and lymph nodes and spleens were taken to analyze DCs activation and T cells ratio. (a) Flow cytometric representative graphs of CD86 and CD80 positive cells in CD11c^+^ DCs in inguinal lymph nodes. (b) Qualification of CD80^+^ or CD86^+^ expressions gated by CD11c^+^ cells according to (a). (c) Flow cytometric representative graphs of CD4 and CD8 positive cells in CD3^+^ T cells of spleen. (d) Qualification of CD4^+^ or CD8^+^ expressions gated by CD3^+^ T cells according to (c) (*n* = 3).

To explore the mechanism on the inhibition of tumor growth by nanovaccine, we evaluated the immune activity *in vivo*. The lymph nodes and spleen of tumor mice were isolated by flow cytometry, and DC activation and T cells expression in the lymph nodes were analyzed. As expected, DCs from OVA-IMQ-VMSNs treated group presented a higher expression level of co-stimulators than that of other groups at the same dose, with 13.40% of CD11c^+^CD86^+^ cells and 19.43% of CD11c^+^CD80^+^ cells (Figure 8a,b). It showed that OVA-IMQ-VMSNs actively induced anti-tumor immune response by promoting DC maturation at lymph nodes.

As is generally accepted that, T cell activation is a prerequisite for its anti-tumor effect. CD8^+^ T cells are also known as cytotoxic T lymphocytes or CTLs and play an important role in anti-tumor. Once CD8^+^ T cells recognize tumor antigens and are activated, it destroys the target cells directly (Feng et al., 2021; Li et al., 2021; Liu et al., 2022). The proportion of CD4^+^ and CD8^+^ T cells in the spleen of tumor bearing mice was also analyzed by flow cytometry, and the results were displayed in Figure 8c,d. After treatment, there was no significant difference in the proportion of CD4^+^ T cells between OVA-IMQ-MSNs group (9.17% of CD3^+^CD4^+^ T cells) and OVA-IMQ-VMSNs group (9.78% of CD3^+^CD4^+^ T cells), which were both higher than that of PBS group and free drugs group. Noteworthy, CD8^+^ T cells in spleen from OVA-IMQ-VMSNs treated group presented a higher expression level than that of other groups at the same dose, with 13.00% of CD3^+^CD8^+^ T cells. It showed that the nanovaccine was able to can improve its anti-tumor effect by promoting the proportion of CD8^+^ T and CD4^+^ T subsets.

Expression of infiltrating T cells was measured in tumor tissues from different treatment groups. Based on immunofluorescence results, OVA-IMQ-VMSNs treated mouse tumor tissues showed the strongest fluorescence signals of CD4^+^ and CD8^+^ T cells, as well as the largest proportion of M1 macrophages (Figure 7f). As expected, it showed that OVA-IMQ-VMSNs nanovaccine could improve its anti-tumor effect by promoting the proportion of CD8^+^ T and CD4^+^ T subsets in tumor tissues, increasing the proportion of M1/M2 macrophages to a certain extent, thereby alleviating the tumor immunosuppressive environment.

### 3.7. Bio-safety evaluation

**Figure 9.**
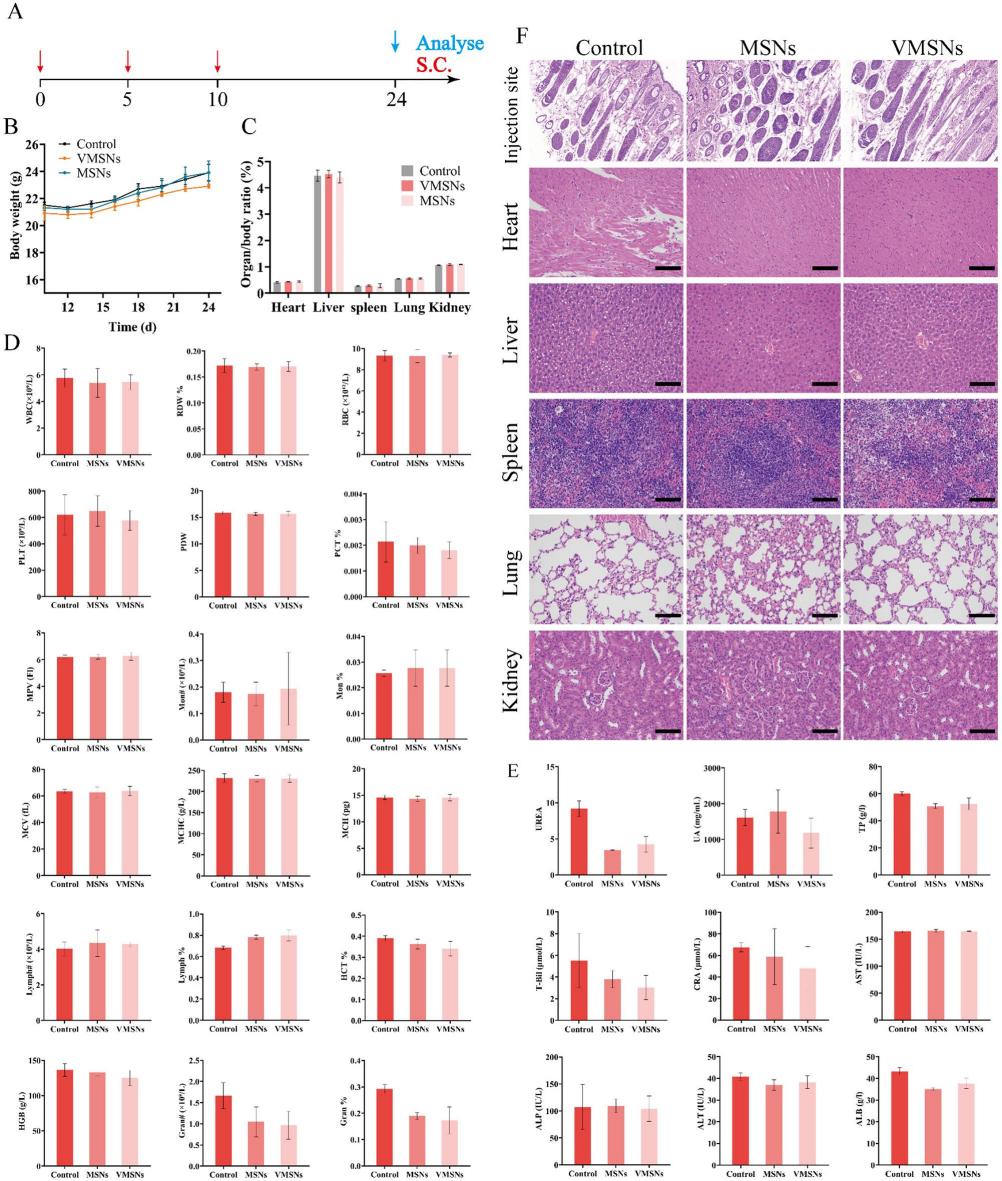
(a) Treatment protocols on the *in vivo* toxicity study of VMSNs and MSNs. (b) Dynamic body weight changes of mice during the treatments. (c–f) Organ/body ratio (c), hematology results (d), biochemical results (e), and representative histopathology images of the major organs and injection site tissue (f) after subcutaneous injection of VMSNs and MSNs. Scale bar: 100 μm.

Finally, we subcutaneously injected VMSNs, MSNs and PBS on days 0,5 and 10. During the experiment, the weight changes of mice were recorded and blood was taken for blood routine and blood biochemical tests after 14 days. After the mice were sacrificed, important organ sections were taken for observation to evaluate the biosafety of the vaccine (Figure 9a). During the experiment, mice in the control group gained 10.0% weight, while mice in the VMSNs and MSNs groups gained 8.7% and 10.9% weight, respectively. There was no weight loss in mice (Figure 9b). As shown in Figure 9d, all hematological parameters of mice treated with VMSNs and MSNs were within the normal range. At the same time, plasma was separated for blood biochemical detection, and plasma albumin (ALB), alanine aminotransferase (ALT), total protein (TP), urea, total bilirubin (*T*-Bil), aspartate aminotransferase (AST), uric acid (UA), creatinine (CRE), alkaline phosphatase (ALP) and other indicators were analyzed. The results showed that the biochemical test results were normal, no significant difference between the experimental group and the control group (Figure 9e). After the animals were sacrificed, the heart, liver, spleen, lung, kidney and other major organs were separated and weighed for histological evaluation. Each organ coefficient index was within the reference range (Figure 9c), and there was no statistical difference between the experimental group and the control group. As shown in Figure 9f, the slice image shown that the tissue structure was complete and clear, and no obvious histological changes, obvious lesions, injuries, inflammation and other histological abnormalities were found. In summary, these results indicated that VMSNs would not produce toxicity after entering the body and had great application potential for diagnosis and treatment.

## 4. Conclusions

Viruses are nanoscale entity with spike-enriched structure and rough surface topology, which can efficiently infect organisms and are regarded as natural immunologic stimulants. Especially in the background of the current Covid-19 pandemic, we are more curious about whether the characteristics of the viruses with such potent infection program can be applied in the design of nanocarriers. Inspired by viruses, we constructed engineered virus-like VMSNs (spherical NPs, negative charge, 60 nm) with spike structure and rough surface topology by mimicking the unique shapes and specific functions of viruses, and served as the nanovaccine to load receptor agonis IMQ and antigen OVA. Encouraging, various experimental results *in vitro* and *in vivo* discovered that virue-like VMSNs do have superior cell invasion ability and could fulfill the delivery task into cells perfectly. That is (1) the unique surface morphology of VMSNs could increase the degree of cell internalization and lysosome escape through unique clathrin/caveolae-independent endocytosis pathway. (2) OVA-IMQ-VMSNs efficiently stimulated DCs cell maturation and cytotoxic T lymphocyte production in the B16-OVA tumor model, thereby inhibiting tumor growth. (3) VMSNs-based nanovaccines not only activated antigen-presenting cells *in vivo*, but also induced tumor immune responses and inhibit tumor growth. (4) VMSNs-based nanovaccine was proven to be biocompatible in both cellular and *in vivo* level, and could meet the requirement of bio-materials in clinic. Therefore, VMSNs has great potential as to function as a vaccine carrier and can better stimulate the immune response compared to its spherical competitor MSN with smooth boundary. This work undoubtedly provides a good basis for further optimizing the nanovaccine delivery system and exploring the effect of nanovaccine surface morphology on the delivery system. We also believe that more VMSNs-based nanovaccines which can be cooperated with the anti-PD-1/L1 antibody or combined with some chemotherapeutic drugs with be synthesized and applied in cancer immunotherapy in the near future.
